# Functional characterization of *Plasmodium berghei* PSOP25 during ookinete development and as a malaria transmission-blocking vaccine candidate

**DOI:** 10.1186/s13071-016-1932-4

**Published:** 2017-01-05

**Authors:** Wenqi Zheng, Fei Liu, Yiwen He, Qingyang Liu, Gregory B. Humphreys, Takafumi Tsuboi, Qi Fan, Enjie Luo, Yaming Cao, Liwang Cui

**Affiliations:** 1 Department of Pathogen Biology, College of Basic Medical Sciences, China Medical University, Shenyang, Liaoning 110001 China; 2 Laboratory of Surgery, The Affiliated Hospital, Inner Mongolia Medical University, Hohhot, 010050 China; 3 Department of Immunology, College of Basic Medical Sciences, China Medical University, Shenyang, Liaoning 110001 China; 4 Department of Entomology, The Pennsylvania State University, University Park, PA 16802 USA; 5 Cell-free Science and Technology Research Center, Ehime University, Matsuyama, Ehime 790-8577 Japan; 6 Dalian Institute of Biotechnology, Dalian, Liaoning China

**Keywords:** *Plasmodium berghei*, PSOP25, Ookinete, Transmission-blocking vaccine

## Abstract

**Background:**

*Plasmodium* ookinete surface proteins as post-fertilization target antigens are potential malaria transmission-blocking vaccine (TBV) candidates. Putative secreted ookinete protein 25 (PSOP25) is a highly conserved ookinete surface protein, and has been shown to be a promising novel TBV target. Here, we further investigated the TBV activities of the full-length recombinant PSOP25 (rPSOP25) protein in *Plasmodium berghei*, and characterized the potential functions of PSOP25 during the *P. berghei* life-cycle.

**Methods:**

We expressed the full-length *P. berghei* PSOP25 protein in a prokaryotic expression system, and developed polyclonal mouse antisera and a monoclonal antibody (mAb) against the recombinant protein. Indirect immunofluorescence assay (IFA) and Western blot were used to test the specificity of antibodies. The transmission-blocking (TB) activities of antibodies were evaluated by the *in vitro* ookinete conversion assay and by direct mosquito feeding assay (DFA). Finally, the function of PSOP25 during *Plasmodium* development was studied by deleting the *psop25* gene.

**Results:**

Both polyclonal mouse antisera and anti-rPSOP25 mAb recognized the PSOP25 proteins in the parasites, and IFA showed the preferential expression of PSOP25 on the surface of zygotes, retorts and mature ookinetes. *In vitro*, these antibodies significantly inhibited ookinetes formation in an antibody concentration-dependent manner. In DFA, mice immunized with the rPSOP25 and those receiving passive transfer of the anti-rPSOP25 mAb reduced the prevalence of mosquito infection by 31.2 and 26.1%, and oocyst density by 66.3 and 63.3%, respectively. Genetic knockout of the *psop25* gene did not have a detectable impact on the asexual growth of *P. berghei*, but significantly affected the maturation of ookinetes and the formation of midgut oocysts.

**Conclusions:**

The full-length rPSOP25 could elicit strong antibody response in mice. Polyclonal and monoclonal antibodies against PSOP25 could effectively block the formation of ookinetes *in vitro* and transmission of the parasites to mosquitoes. Genetic manipulation study indicated that PSOP25 is required for ookinete maturation in *P. berghei*. These results support further testing of the PSOP25 orthologs in human malaria parasites as promising TBV candidates.

**Electronic supplementary material:**

The online version of this article (doi:10.1186/s13071-016-1932-4) contains supplementary material, which is available to authorized users.

## Background

Malaria remains one of the most prevalent tropical infectious diseases and is endemic in nearly 95 countries and territories around the world, with estimated > 3.2 billion people being at risk. In 2015, there were approximately 214 million new malaria cases resulting in 438,000 deaths, ~ 80% of which occurred in Africa [1]. Currently, due to the spread of insecticide-resistant mosquitoes and multidrug-resistant parasites, major malaria control efforts including vector control and chemotherapy are becoming increasingly ineffective [2–4]. These trends highlight the need for developing an integrated malaria control strategy to eliminate malaria transmission. A transmission-blocking vaccine (TBV) targeting the sexual stages of the *Plasmodium* has the potential to reduce malaria transmission and prevent the spread of resistant parasites. It is predicted that TBV administration can reduce child mortality even in areas of high endemicity [5]. Additionally, TBV can slow down the spread of mutant parasites, which will prolong the effective lives of antimalarial drugs and vaccines [6]. Mathematical models further predict that TBVs will be an effective tool for malaria elimination [7].

TBV is designed to target the *Plasmodium* antigens expressed during sexual development or *Anopheles* midgut proteins that interact with sexual stages and allow ookinetes to traverse the *Anopheles* midgut epithelial cells. Research on TBVs has led to the identification and experimental validation of several potential TBV candidates, but only a few including Pfs48/45 [8, 9], Pfs230 [10, 11] and Pfs25 [12] in *P. falciparum*, and Pvs25 and Pvs28 in *P. vivax* [13], have been found effective in blocking parasite transmission. Investigations on the two 6-cysteine domain protein family members, Pfs48/45 and Pfs230, have shown that anti-Pfs48/45 monoclonal and polyclonal antibodies in experimental animals can effectively inhibit the transmission of *P. falciparum* to mosquitoes [9, 14, 15], while Pfs230-raised antibodies are sufficient to block development of the oocysts and competent to induce complement-dependent transmission-blocking (TB) activity [11]. Furthermore, antibodies against both Pfs48/45 and Pfs230 have been detected in natural infections, thereby bringing the potential to boost and/or enhance antibody titers with TBVs against these antigens [16]. Unlike pre-fertilization proteins, post-fertilization antigens are expressed solely after the formation of the zygotes within the mosquito midgut. Concealed from the host’s immune system, these antigens have limited diversity among the parasite populations [17, 18]. The major ookinete surface protein Pfs25 is a well-characterized 25-kDa glycosyl-phosphatidylinositol (GPI)-anchored protein with four epidermal growth factor-like domains. Pfs25 is involved in adhesion of ookinete and plays an important role in subsequent penetration of the mosquito midgut [19, 20]. Mouse antiserum against native Pfs25 [21], heterologously expressed Pfs25, or the *P. vivax* ortholog Pvs25 proteins can effectively inhibit parasite development in mosquitoes [22–24]. Though Pfs25 and Pvs25 provide evidence for the efficacy of post-fertilization antigens in TBVs, more TBV candidate antigens and higher levels of TB activities are needed for an effective deployable vaccine.

With efforts for identifying new TBV candidates, we have recently identified a post-fertilization antigen PSOP25 (PBANKA_111920) in the rodent parasite *Plasmodium berghei. Psop25* encodes a 350 amino acid (aa) protein with a signal peptide, and the native protein is predicted to be 40 kDa. *Psop25* transcript is highly expressed in ookinetes and occupied in the 99th percentile in the transcriptome of ookinetes [25]. Ookinete-specific expression of this protein was confirmed in our previous study [26]. Antisera from mice immunized with a partial PSOP25 domain (aa 45–245), which included ten predicted antibody epitopes, inhibited ookinete formation by 53.0% in *in vitro* ookinete cultures. Mosquitoes fed on this partial PSOP25 domain-immunized mice also resulted in modestly decreased oocyst prevalence (25.0%) and significantly reduced oocyst densities (64.3%) [26], suggesting that PSOP25 could be a new promising target for TBVs. Here we set out to further investigate the TBV activities of the full-length PSOP25 protein in *P. berghei*, and characterize the functions of PSOP25 by genetic knockout (KO).

## Methods

### Mice, parasites and mosquitoes

Female BALB/c mice (six- to eight-week-old; Beijing Animal Institute, Beijing, China) were used for all animal experiments. *P. berghei* (ANKA strain 2.34) and Δ*psop25* lines (*psop25* gene knockout line) were maintained in mice and used for challenge infection. Adult *Anopheles stephensi* mosquitoes of the Hor strain were fed with 10% (w/v) glucose solution and maintained in an insectary with a surrounding of 50–80% relative humidity, at 25 °C.

### Expression and purification of rPSOP25

For the expression of full-length PSOP25, a *psop25* fragment encoding aa 25–350 (excluding the signal peptide) was amplified from *P. berghei* genomic DNA with *psop25*-F and *psop25*-R primers (Additional file 1: Table S1). The *psop25* fragment and the prokaryotic expression vector pET30a (+) (Novagen, Darmstadt, Germany) were digested with restriction enzymes *Nde*I and *Hind*III, then ligation was performed using the Ligation High Kit (Toyobo, Osaka, Japan). The recombinant plasmid was transformed in *Escherichia coli* BL-21 (Novagen, Darmstadt, Germany) and the His-tagged recombinant PSOP25 (rPSOP25) was expressed at 20 °C for 12 h after induction with 1 mM isopropyl-β-D-thiogalactopyranoside (Sigma-Aldrich, St. Louis, USA). For protein purification, cultures were harvested and lysed using binding buffer containing 10 mM imidazole, 300 mM NaCl and 50 mM sodium phosphate (pH 8.0) and treated by sonication (15 cycles of 20 s pulses and 30 s intervals). The soluble rPSOP25 was purified by Ni-NTA His-Bind Superflow (Novagen, Darmstadt, Germany), according to the manufacturer’s instructions. Purified rPSOP25 was extensively desalted in 0.1 M phosphate buffered saline (PBS, pH 7.4) overnight at 4 °C, and then analyzed by SDS-PAGE.

### Animal immunization and monoclonal antibody (mAb) production

To obtain polyclonal antisera against rPSOP25, a group of six female BALB/c mice were subcutaneously immunized with the purified protein (50 μg/mouse) emulsified in complete Freund’s adjuvant (Sigma-Aldrich, St. Louis, USA), which has been used to produce high-titer antibodies [27]. Subsequently, the mice were given two booster immunizations of 25 μg of rPSOP25 at 3-week intervals with the rPSOP25 protein emulsified in incomplete Freund’s adjuvant (Sigma-Aldrich, St. Louis, USA). A group of negative control mice (*n* = 6) were immunized with PBS and same adjuvant formulations. For the final bleed, mouse blood was collected at 10 days after the final immunization by cardiac puncture and the antisera were obtained after the blood had clotted at room temperature. Antisera from individual mice were mixed together and used in the subsequent trials.

For monoclonal antibody (mAb) production, rPSOP25-immunized BALB/c mice were obtained as described above, then the spleen cells of the immunized mice were extracted and fused with Sp2/0-Ag14 myeloma cells to produce the anti-PSOP25 mAb [28]. The fused hybridoma cells were generated using the traditional polyethylene glycol method, and then selected in the hypoxanthine-aminopterin-thymidine medium. The antibodies were screened by indirect antibody capture enzyme-linked immunosorbent assay (ELISA). The IgG fractions were prepared by ammonium sulfate precipitation, and then purified on a Protein A column (ThermoFisher Scientific, Waltham, USA), according to the manufacturer’s instructions. The mAb isotype was determined by using the SBA Clonotyping™ System-HRP (Southern Biotechnology Associates, Birmingham, USA).

### ELISA

Antibody titers to rPSOP25 were determined by ELISA on day 14, 35 and 52 after the first immunization as previously described [26]. Briefly, 96-well plates were coated overnight with purified rPSOP25 at 4 °C, and blocked with blocking buffer (0.05% Tween 20 in 0.1 M PBS, 1% bovine serum albumin, pH 7.4) for 2 h at 37 °C. The plates were then washed twice with PBS-T (0.05% Tween 20 in 0.1 M PBS, pH 7.4) and incubated with pooled mouse anti-rPSOP25 sera (1:200 dilution) in blocking buffer at 37 °C for 2 h. After two washes, the wells were incubated for 2 h at 37 °C with a 1:5000 dilution of HRP-conjugated goat anti-mouse IgG antibody (Invitrogen, Waltham, USA). After five final washes, tetramethyl benzidine (Amresco, Solon, USA) was added and the reaction was stopped by 2 mM H_2_SO_4_. The absorbance at 490 nm was measured with an ELISA plate reader.

For estimating the end point titer of immunized mice, sera from all mice in each immunization and control group were pooled and diluted from 1:200 to 1:204800 in a blocking buffer and incubated at 37 °C for 2 h. The end point titers of the total IgG corresponded to the highest dilution at which the OD490 value was higher than the cut-off value, which was defined as the mean of the pooled negative control antisera + 3 × standard deviation [29].

### Ookinete enrichment and lysate preparation

The enrichment of ookinetes was referred to a modified protocol [30]. Briefly, 1.2 mg phenylhydrazine (Sigma-Aldrich, St. Louis, USA) in 0.9% NaCl were intraperitoneally (i.p.) injected into BALB/c mice 3 days before *P. berghei* infection. These treated mice were then i.p. injected with 5 × 10^6^ *P. berghei*-infected red blood cells (iRBCs) to initiate the blood-stage infection. Parasitemia was allowed to reach approximately 1–3% at three days post-infection (p.i.), when the mice were anesthetized. After removal of the white blood cells, the infected blood was diluted 1:10 with the ookinete culture medium (100 mg/l neomycin, 50 mg/l streptomycin, 50 mg/l penicillin, 20% (v/v) FBS, and 1 mg/l heparin in RPMI 1640, pH 8.3) in a petri dish and maintained at 19 °C for 24 h. The culture was then diluted in 45 ml of 0.17 M NH_4_Cl on ice for 10 min to lyse erythrocytes. After a wash with 0.1 M PBS, ookinetes were separated on a 62% (v/v) Nycodenz/PBS cushion by centrifugation (1300× *g*) for 25 min at 25 °C, treated with 0.15% saponin and washed once with 0.1 M PBS. The ookinete lysate was prepared by resuspending the ookinetes in 2% SDS containing 1% Triton X-100 and 1 × protease inhibitor cocktail (Roche, Castle Hill, Australia) for 30 min at room temperature.

### Western blot

The parasite antigens (10 μg) or purified rPSOP25 (500 ng) were subjected to electrophoresis under reducing or non-reducing conditions using a 10% SDS-PAGE gel and electro-transferred to PVDF membrane (Bio-Rad, Hercules, USA). Western blot was performed essentially as described [26]. Primary antibodies were the pooled mouse anti-rPSOP25 serum (1:200) or anti-rPSOP25 mAb (1:1000), and HRP-conjugated goat anti-mouse IgG antibodies (1:10,000) (Invitrogen, Waltham, USA) were used as the secondary antibodies. Pbs21 mAb (clone 13.1, 1:1000) was included as a positive control [31]. The sera (1:200) obtained from mice immunized with the PBS-adjuvant formulations were used as the negative control. The blot was developed using an ECL Western Blotting Kit (ThermoFisher Scientific, Waltham, USA).

### Indirect immunofluorescence assay (IFA)

Parasites containing asexual stages, gametocytes, zygotes and ookinetes of *P. berghei* were fixed on slides [32]. Anti-rPSOP25 mAb (1:500) or Pbs21 mAb (clone 13.1, 1:500, positive control) or negative control sera (1:500) was incubated in 5% skim milk and labeled with FITC-conjugated goat anti-mouse IgG (1:500; Invitrogen, Waltham, USA) at 37 °C for 1 h. After staining of nuclei with 4′, 6-diamidino-2-phenylindole (DAPI; Invitrogen, Waltham, USA), the slides were examined under Olympus BX53 (Olympus Corporation, Center Valley, USA) and the images were processed using Adobe Photoshop (Adobe Systems Inc., San Jose, USA).

### Quantification of TB activities

For the *in vitro* assay, phenylhydrazine pre-treated mice were infected as described above. On day 3 p.i., parasitemia was determined, and 10 μl of blood were taken from appropriate hosts and added to 90 μl ookinete culture medium containing anti-rPSOP25 serum or negative control mouse serum at final dilutions of 1:5, 1:10, and 1:50. Additionally, anti-rPSOP25 mAb was added to the ookinete culture at 10, 5 and 1 μg/100 μl (concentration of mAb was 0.5 μg/μl) of ookinete culture, respectively. Ookinete cultures were incubated at 19 °C for 24 h and the ookinete conversion rates were determined as described previously [26, 32].

For direct mosquito feeding assays (DFA), experimental mice (*n* = 3) were immunized with rPSOP25 and negative control mice (*n* = 3) were immunized with the PBS-adjuvant formulations as described above. Ten days after the final immunization, mice were infected i.p. with 5 × 10^6^ *P. berghei* ANKA iRBC. For the antibody transfer experiment, three normal mice were injected intravenously with 150 μg of anti-rPSOP25 mAb/mouse 1 h before mosquito feeding. Four-day-old female *A. stephensi* mosquitoes (starved for 12 h) were allowed to feed on rPSOP25 immunized mice or antibody-transferred mice for 30 min. Engorged mosquitoes were maintained in an insectary at 21 °C and 70% relative humidity. Ten days after feeding, ~ 30 mosquitoes were dissected, and midguts were stained with 0.5% mercurochrome (Sigma-Aldrich, St. Louis, USA) to count the number of oocysts per midgut.

### Generation of *psop25* KO parasites

To knock out all the protein-coding sequence of the *psop25 g*ene, the target vector containing an *hdhfr* selection cassette was used (kindly provided by plasmoGEM, vector design ID, PbGEM-042760; http://plasmogem.sanger.ac.uk/). Before *P. berghei* transfection, vector DNA was digested by *Not*I followed by ethanol precipitation. The linearized plasmid (10 μg) was electroporated into purified schizonts using the Nucleofector device as described previously [32]. After transfection, the complete parasite suspension was injected intravenously *via* the tail vein into mice. Following a 24 h incubation period, infected mice were treated for 3–4 days with pyrimethamine (Sigma-Aldrich, St. Louis, USA) *via* drinking water (70 μg/ml). Infected blood was collected and confirmed by integration-specific PCR (Additional file 1: Table S1). Monoclonal parasite lines were then obtained by limiting dilution.

### Phenotypic analysis of the Δ*psop25* line

To study whether deletion of *psop25* affected parasite growth, five phenylhydrazine-treated mice were inoculated i.p. with either 5 × 10^6^ wildtype (WT) or Δ*psop25* iRBCs. For each genotype, blood smears were used to monitor daily parasitemia, gametocytemia (mature gametocytes per 100 RBCs), and the gametocyte sex ratio (female: male ratio) [33]. To quantify male gamete exflagellation, 10 μl of *P. berghei*-infected blood collected from mouse tail vein on day 3 p.i. were added into 90 μl of ookinete culture medium and incubated for 15 min at 25 °C, and the exflagellation centers were counted as previously described [32]. At the same time, ookinetes were cultured *in vitro* as described above [34], and the ookinete conversion rates were determined by IFA using a Pbs21 mAb [35]. For oocyst quantifications, mice at 3 days p.i. with each parasite line were fed to starved *A. stephensi* mosquitoes for 30 min [36, 37]. Ten days after feeding, ~ 30 fed mosquitoes from each genotype were dissected for counting of the number of oocysts per infected mosquito and to determine the prevalence and intensity of infection.

### Statistical analysis

Parasitemia, gametocytemia, gametocyte sex ratio and ookinete conversion rates between groups were analyzed by the Student’s *t*-test, using GraphPad Prism software. The prevalence of infection (proportion of infected mosquito) was analyzed by the Fisher’s exact test, while the intensity (number of oocysts per midgut) was analyzed by the Mann-Whitney *U-*test [38], using SPSS version 17.0. *P*-values less than 0.05 were considered statistically significant.

## Results

### The full-length rPSOP25 is immunogenic

**Fig. 1:**
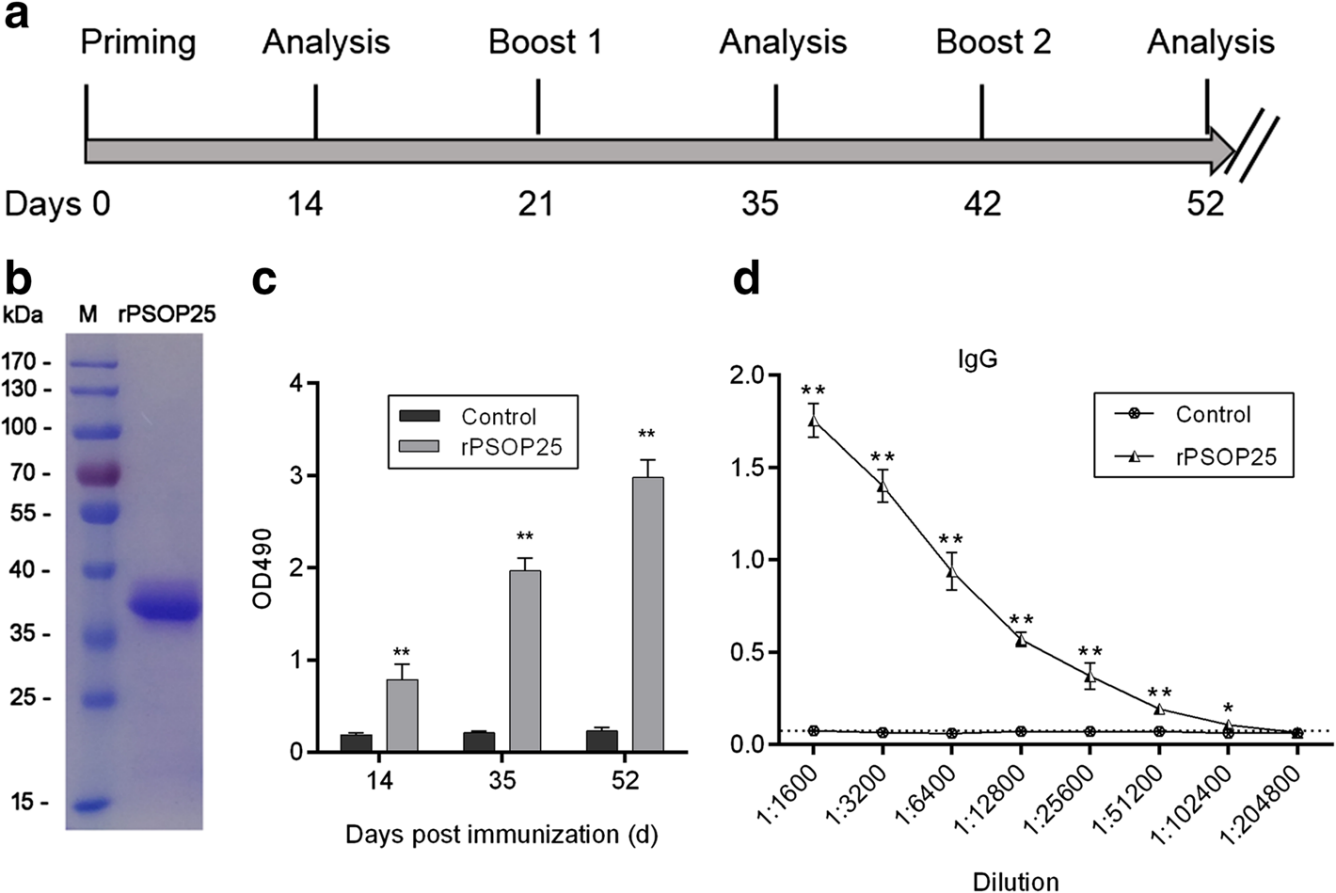
**a** Mouse immunization and analysis scheme. **b** rPSOP25 was purified from *E. coli* and analyzed on a 10% SDS-PAGE gel. **c** Antibody titers in immunized mice during immunization, experimental mice were immunized with rPSOP25 formulated in Freund’s adjuvant; control mice were immunized with only Freund’s adjuvant and 0.1 M PBS. The data represent two separate experiments. Error bar shows mean ± standard deviation (SD). SD indicates the assay variance. **P < 0.01 (Student’s *t-*test). **d** Anti-rPSOP25 total IgG titer at 10 days after the final immunization analyzed by ELISA. Mean of control antisera + 3 × SD is shown by the broken lines. IgG titers correspond to the last dilution of the anti-rPSOP25 sera where in OD490 values were above the cut-off values. Cut-off value was defined as that of the pooled sera from control mice. The experiment was performed three times. Error bar shows mean ± SD. *P < 0.05, **P < 0.01 (Student’s *t-*test). *Abbreviations*: M, molecular weight marker; rPSOP25, purified rPSOP25 under reducing conditions

A PSOP25 fragment which corresponded to aa 25–350 excluding the signal peptide was expressed in *E. coli.* This fragment included 14 predicted B cell epitopes [39] (Additional file 2: Figure S1). The purified rPSOP25 protein had a molecular size of ~ 36.5 kDa from SDS-PAGE analysis, which was consistent with the predicted size of PSOP25 protein (Fig. 1a). To determine the immunogenicity of purified rPSOP25, we performed ELISA using pooled serum generated from mice immunized with the recombinant protein. The results showed that immunization with rPSOP25 induced strong antibody responses as compared to the negative control; the rPSOP25-specific IgG titers increased continuously during the course of vaccination (Student’s *t-*test: *t*_(10)_ = 35.13, *P <* 0.0001; Fig. 1b). The antisera collected 10 days after the final immunization with rPSOP25 reached a titer of 1:1024000 (Fig. 1c). Meanwhile, an anti-rPSOP25 mAb was produced from a selected hybridoma line, which was determined to be the IgG1 isotype (Additional file 2: Figure S2).

### Anti-rPSOP25 antisera and mAb recognize the ookinete proteins

**Fig. 2:**
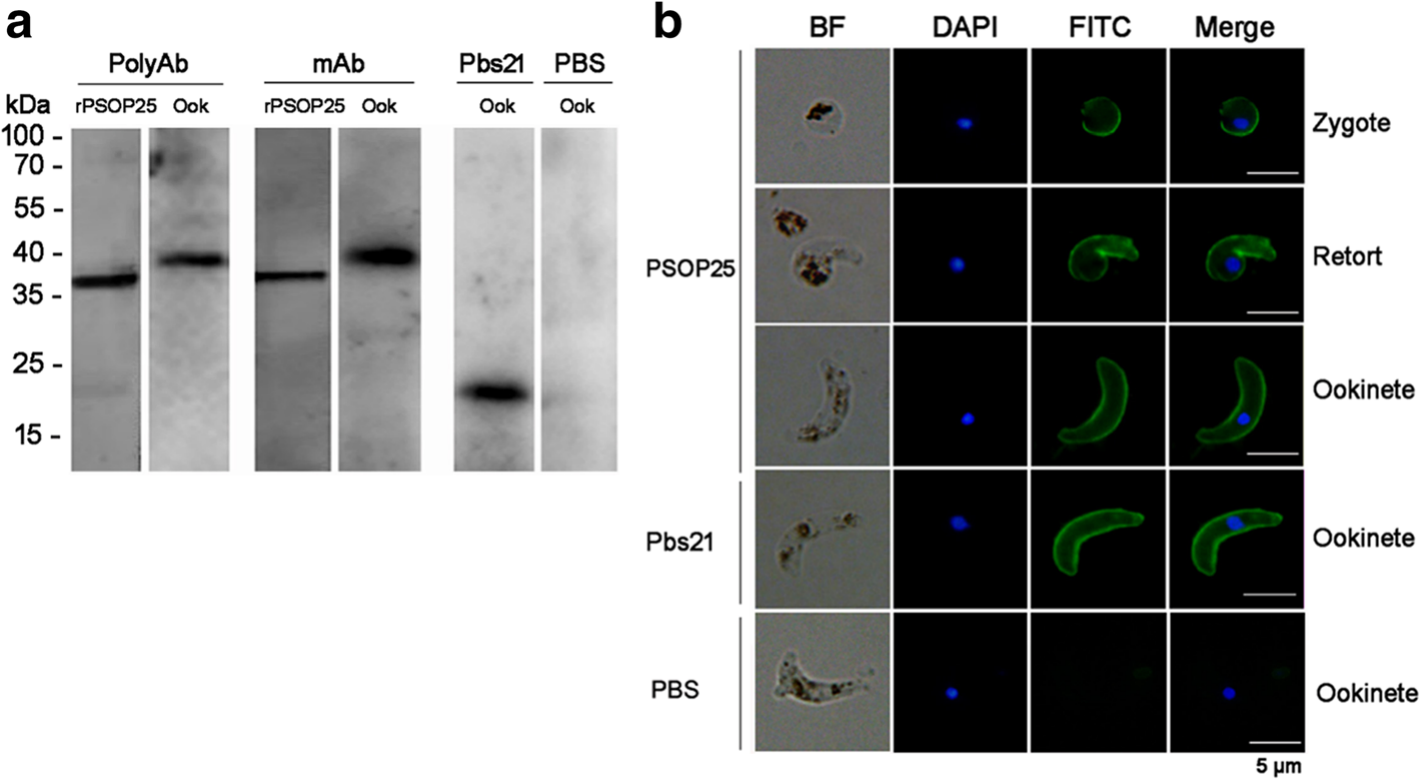
**a** Western blot analysis of purified rPSOP25 protein and *P. berghei* ookinete (Ook) lysates with anti-rPSOP25 sera (PolyAb) and anti-rPSOP25 mAb. Lysates were subjected to electrophoresis under reducing conditions by SDS-PAGE. Pbs21 mAb was used as positive control; a control mouse serum (PBS) was used as negative control. **b** IFA was performed on zygote, retort and ookinete at different time points of ookinete culture using anti-rPSOP25 mAb (green - FITC). Positive control - Pbs21 mAb, negative control - a control mouse serum (PBS). Nuclei were labelled with DAPI (blue). BF, bright field. *Scale-bars*: 5 μm

The specificity of the pooled anti-rPSOP25 antisera and mAb was determined by Western blot against the rPSOP25 or protein lysate from ookinetes. On Western blots, both the antisera and mAb detected the 36.5 kDa rPSOP25 (Fig. 2a) and a band of approximately 40 kDa in the lysate of purified ookinetes under reducing conditions (Fig. 2a) and non-reducing conditions (Additional file 2: Figure S3), which is close to the predicted size of PSOP25. In a previous study, IFA using antisera raised against a partial domain of the PSOP25 protein indicated that PSOP25 is expressed on the surface of ookinetes [26]. Consistently, IFA with the anti-rPSOP25 mAb using zygotes, retorts and ookinetes without membrane permeabilization revealed strong fluorescence of the parasite body, which agrees with the surface localization of PSOP25 (Fig. 2b).

### Antibodies against PSOP25 show obvious TB activities

**Fig. 3:**
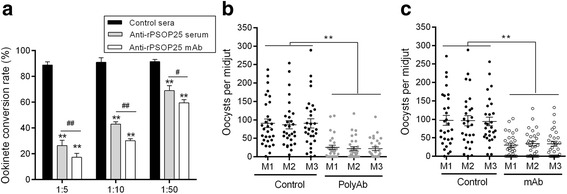
**a** TB activities of anti-rPSOP25 serum and anti-rPSOP25 mAb on *P. berghei* ookinete formation *in vitro*. Anti-rPSOP25 serum, anti-rPSOP25 mAb, or normal mouse serum (control) were diluted at 1:5, 1:10 and 1:50, respectively. Means were representative of three independent experiments. Error bar shows mean ± SD. ** indicate significant difference compared with the control sera (*P* < 0.01). # indicate significant difference between anti-rPSOP25 serum and mAb group (*P* < 0.05), ## *P* < 0.01 (Student’s *t*-test). **b** Direct mosquito feeding assay to assess the TB activity of polyclonal antisera in rPSOP25-immunized mice (3 mice per group). **c** Passive antibody transfer experiment to assess the TB activity of the anti-PSOP25 mAb. For b and c, mosquito midguts were dissected at ten days post-infection, the number of oocysts was counted under a microscopy. The data are collated from three experiments. The mean number of oocysts and the SEM in each group are shown. **P < 0.01 (Mann-Whitney *U-*test)

Anti-rPSOP25 antisera and mAb were used in ookinete conversion assay to study the TB activity of the antibodies against PSOP25. When incubated with pooled mouse anti-rPSOP25 antisera or mAb, ookinete conversion was inhibited in a dose-dependent manner. In ookinete cultures supplemented with the pooled immune sera at 1:5, 1:10 and 1:50 dilutions, ookinete conversion rates were reduced by 62.5% (Student’s *t-*test: *t*_(4)_ = 22.52, *P <* 0.0001), 47.9% (Student’s *t-*test: *t*_(4)_ = 21.44, *P <* 0.0001), and 22.5% (Student’s *t*-test: *t*_(4)_ = 9.11, *P =* 0.0008), respectively (Fig. 3a). Compared with the control sera, ookinete conversion rates in cultures with mAb added at 10, 5 and 1 μg/100 μl were reduced by 71.6% (Student’s *t-*test: *t*_(4)_ = 32.04, *P <* 0.0001), 60.8% (Student’s *t*-test: *t*_(4)_ = 27.84, *P <* 0.0001) and 32.0% (Student’s *t*-test: *t*_(4)_ = 18.60, *P <* 0.0001), respectively (Fig. 3a).

To further examine the TB effect of anti-rPSOP25 antibodies *in vivo*, mice were immunized with rPSOP25 or passively transferred with the mAb and used in DFA. Ten days after feeding, mosquitoes were dissected and midgut oocysts were counted. Mosquitoes fed on the rPSOP25-immunized mice showed a 31.2% reduction in the prevalence of oocysts, as compared to the control groups. The mean prevalence was 68.7% in mosquitoes fed on the rPSOP25-immunized mice, whereas it was 99.9% in mosquitoes fed on the control mice (Fisher’s exact test: OR = 40.72, 95% CI = 5.38–307.91, *P* < 0.001; Fig. 3b, Table 1). Moreover, mosquitoes fed on rPSOP25-immunized mice revealed a 66.3% reduction in oocyst density compared to the control group (Mann-Whitney *U-*test: *Z* = -8.32, *P* < 0.0001; Fig. 3b, Table 1). Similarly, mosquitoes fed on the mice passively transferred with the mAb against PSOP25 had a 26.1% reduction in the prevalence of oocysts (Fisher’s exact test: OR = 16.46, 95% CI = 3.76–72.13, *P* < 0.001; Fig. 3b, Table 1) and a 63.3% reduction in density of oocysts (Mann-Whitney *U-*test: *Z* = -6.97, *P* < 0.0001; Fig. 3b, Table 1).

**Table 1:** Evaluation of TB effect of anti-rPSOP25 serum and mAb in mosquito feeding assays

	Immunization groups^a^						Monoclonal antibody transfer groups^b^					
	Control mice			rPSOP25 immunized mice			Control mice			mAb transferred mice		
	M1	M2	M3	M1	M2	M3	M1	M2	M3	M1	M2	M3
No. of mosquitoes infected/dissected	30/30	30/30	29/30	20/28	20/30	19/28	28/29	27/28	28/28	22/30	21/29	20/29
Prevalence of infection (%)^c^	100	100	99.7	71.4	66.7	67.9	96.6	96.4	100	73.3	72.4	69.0
Mean prevalence (%)	99.9			68.7*					97.7			71.6*
Reduction in prevalence (%)^d^						31.2						26.1
Oocyst intensity^e^	90.7	86.8	90.5	25.2	22.1	21.7	97.1	96.3	94.3	29.8	34.2	33.7
SEM^f^	11.58	11.17	12.50	5.890	5.232	5.138	13.47	13.66	11.82	5.462	6.373	6.800
Mean oocyst intensity			89.3			23.0*			95.9			32.6*
Reduction in oocyst intensity (%)^g^						66.3						63.3

^a^TB activity assay was carried out using rPSOP25-immunized mice

^b^TB activity assay was carried out using BALB/C mice transferred with the anti-PSOP25 mAb

^c^The prevalence of infection was calculated by the number of mosquitoes with oocysts/total mosquitoes dissected in each group × 100%

^d^The percent reduction of prevalence was calculated as % mean prevalence _control_ – % mean prevalence _PSOP25_

^e^Mean number of oocysts per mosquito midgut

^f^Standard error of the mean

^g^The percent reduction in oocyst intensity was calculated as (mean oocyst intensity _control_ – mean oocyst intensity _PSOP25_)/mean oocyst intensity _control_ × 100%

*P < 0.001 for comparisons between the experimental group and the control group

### PSOP25 is required for the maturation of ookinetes

**Fig. 4:**
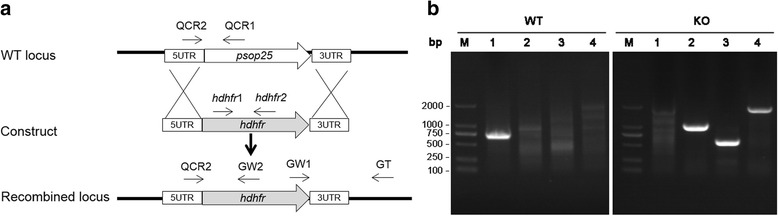
**a** Schematic representation of the WT locus, the construct used for transfection and the recombined locus with *psop25* replaced with the *hdhfr* cassette. Primers QCR1, QCR2, GW2, *hdhfr*1, *hdhfr*2, GW1 and GT used for diagnostic PCR of the WT locus or knockout are marked. **b** Lane 1: primers QCR1 + QCR2 (696 bp) are used for diagnostic PCR of the WT locus. Lanes 2, 3 and 4 are PCR products from primers GW2 + QCR2 (1,002 bp), *hdhfr*1 + *hdhfr*2 (561 bp), GW1 + GT (1,831 bp) for PCR verification of *psop25* KO, respectively

**Fig. 5:**
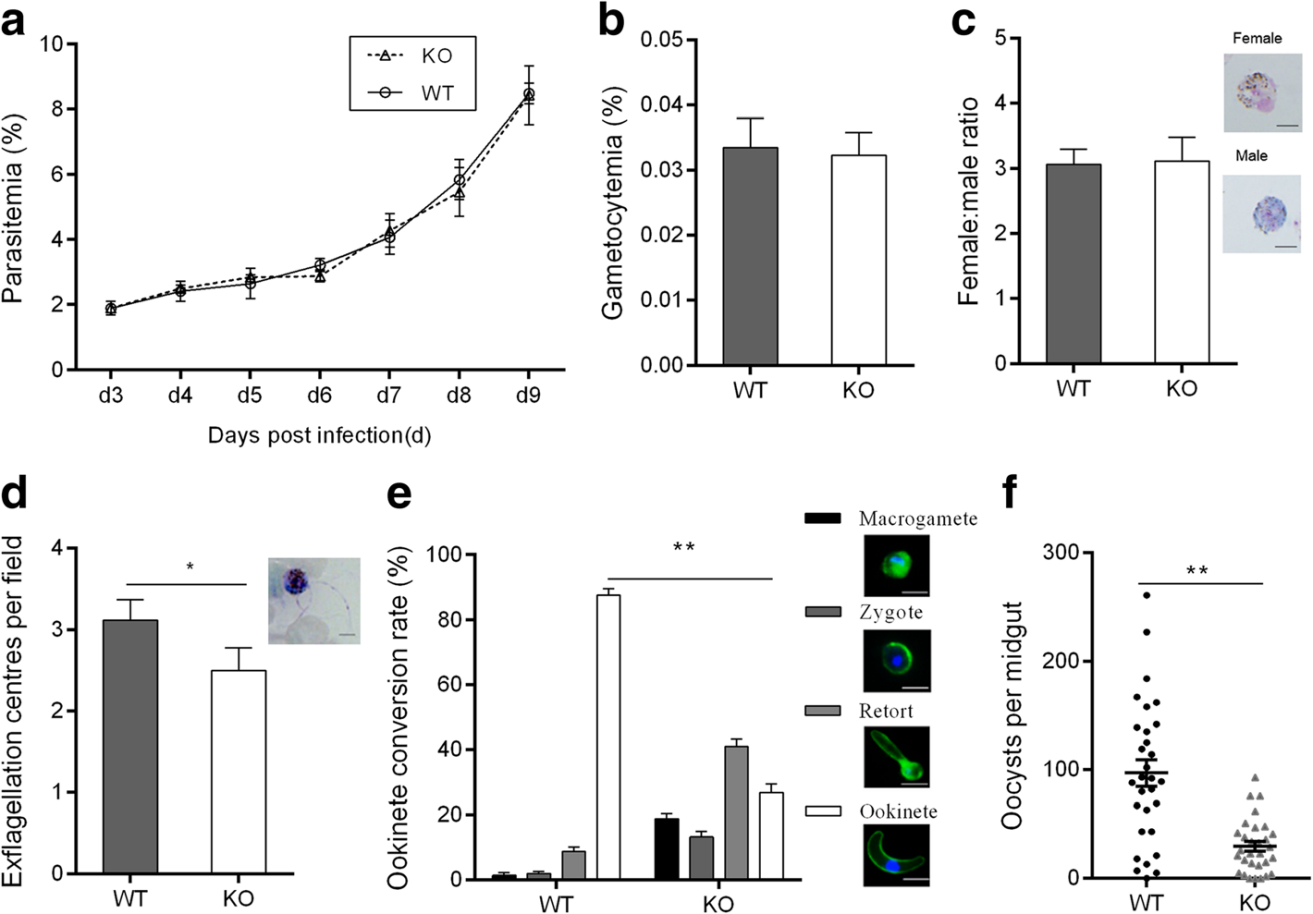
**a** Average parasitemia was calculated in mice after infection with the wild-type (WT) or Δ*psop25* parasites (*n* = 5). **b** Gametocytemia in mice infected with WT or Δ*psop25* parasites (*n* = 3). **c** Female: male gametocyte ratios of WT or Δ*psop25* parasites (*n* = 3). **d** Exflagellation of WT and Δ*psop25* microgametes (*n* = 3), *P < 0.05 (Student’s *t-*test). **e** Ookinete conversion rates *in vitro* of WT and Δ*psop25* parasites. For **c**, **d** and **e**, characteristic morphologies of parasites are shown on the right (*n* = 3), **P < 0.01 (Student’s *t-*test). **f** Oocyst number per midgut in mosquitoes 10 days after feeding on mice infected with the WT and the Δ*psop25* parasites. The horizontal bar shows the mean number of oocysts per midgut in mosquito (± SEM). **P < 0.01 (Mann-Whitney *U*-test). For **a**-**f**, all the data are representative of three separate experiments. *Scale-bars*: 5 μm

To determine the function of PSOP25 during *Plasmodium* development, a *psop25* gene KO line (Δ*psop25*) was generated in *P. berghei* (Fig. 4a) [40]. Genotypes of the cloned pyrimethamine-resistant parasites were confirmed by integration-specific PCR (Fig. 4b). To determine if *psop25* gene knockout led to any deficiencies in parasite development, we compared parasitemia, gametocytemia and gametocyte sex ratio between groups of BALB/c mice infected with 5 × 10^6^ Δ*psop25* or WT *P. berghei* parasites. Consistent with no expression of the PSOP25 protein in asexual erythrocytic stages, Δ*psop25* had no evident effect on asexual parasitemia (Fig. 5a). In addition, on day 3 p.i., gametocytemia and gametocyte sex ratio did not differ significantly between the WT parasite and the Δ*psop25* line (Fig. 5b, c). However, mean male gamete exflagellation events were slightly but significantly reduced in the Δ*psop25* line, as compared to the WT parasites (Student’s *t*-test: *t*_(10)_ = 4.01, *P =* 0.0024; Fig. 5d). Furthermore, *in vitro* ookinete cultures established from parasites at day 3 p.i. showed that ookinete conversion rate was significantly reduced in the Δ*psop25* line, although the mature ookinetes in the Δ*psop25* line appeared morphologically normal (data not shown). Using reverse-transcriptase-PCR, we determined that there was no *psop25* expression in the ookinetes of the Δ*psop25* line, further confirming that *psop25* was deleted (data not shown). In the WT line, the zygote, retort, and ookinete conversion rates was 2.0, 8.8 and 87.74%, respectively. Whereas in the Δ*psop25* line, 13.3 and 41.0% parasites progressed to the zygote and retort stages, respectively, further maturation to ookinetes was reduced by 60.9% (Student’s *t*-test: *t*_(4)_ = 31.69, *P <* 0.0001; Fig. 5e), indicating that PSOP25 might play a crucial role in ookinete maturation. The oocyst density was reduced to 29.7 per mosquito midgut in those fed on the Δ*psop25* parasites as compared to 96.9 in WT parasites, reflecting a 69.4% reduction (Mann-Whitney *U-*test: *Z* = -4.25, *P* < 0.0001; Fig. 5f, Table 2).

**Table 2:** Oocyst number per midgut in mosquitoes 10 days after feeding on mice infected with the WT and the Δ*psop25* parasites

	No. of mosquitoes (infected/dissected)	Prevalence of infection (%)^a^	Mean oocyst (IQR)^b^	SEM^c^	Reduction in oocyst intensity (%)^d^	*P*
Wild type	29/30	99.7	96.93 (43.00–139.8)	12.01		
Δ*psop25*	27/30	90	29.67 (10.25–43.25)	4.481	67.26	< 0.001

^a^The prevalence of infection was calculated by the number of mosquitoes with oocysts/total mosquitoes dissected in each group × 100%

^b^IQR, inter-quartile range

^c^Standard error of the mean

^d^The percent reduction in oocyst intensity was calculated as (mean oocyst intensity _WT_ – mean oocyst intensity _Δ*psop25*_)/mean oocyst intensity _WT_ × 100%

## Discussion

Disrupting the parasite life-cycle to prevent the disease from being transmitted to other individuals represents a key component of an integrated malaria control strategy [41]. Despite investigations on several TBV antigens over the last 40 years, there is still a need to discover new TBV vaccine candidates for malaria elimination purpose [42]. In our previous study, we evaluated the transmission-blocking activities of a partial 200 aa PSOP25 domain [26]. Here we expressed the full-length rPSOP25 protein and raised polyclonal antisera as well as mAb for this protein, which were found to possess effective TB activities in an *in vitro* ookinete formation assay and *in vivo* DFA.

Antibody concentrations against TBV candidates, as measured by conventional ELISA, have been shown to be associated with the effectiveness of TB activities in mosquito membrane feeding assays [19, 43]. In our previous study, the 200 aa PSOP25 fragment including ten predicted antibody epitopes had elicited obvious antibody responses, and immunized mouse antisera produced TB activities with 25% reduction in prevalence and 64.3% reduction in oocyst density [26]. The full-length PSOP25 is predicted to contain four additional antibody epitopes, and the full-length rPSOP25 indeed induced high antibody titers in mice. In parallel comparison experiments, mosquitoes fed on mice immunized with the full-length and partial rPSOP25 showed similar levels (~60%) of reduction in oocyst density as compared to those fed on control mice. However, there was a greater degree of reduction in oocyst prevalence in mosquitoes fed on mice immunized with the full-length protein (31.2%) as compared to that in mosquitoes fed on mice immunized with the partial rPSOP25 (25%) [26]. The TB activities of PSOP25 were comparable to those of PSOP12 [44], PSOP7 and PSOP26 [26] in the reduction of oocyst density and infection prevalence in DFA. Furthermore, monoclonal antibodies have been a valuable tool for the characterization of TBVs [45, 46]. Previous studies have explored passive transfer of transmission blocking mAbs (e.g. Pbs21 mAb clone 13.1) for TB activities [31, 47]. In this study, an IgGl-type mAb against PSOP25 significantly inhibited the development of ookinetes and oocyst when administered through passive transfer prior to mosquito feeding, and the TB activities were comparable to those from the full-length rPSOP25 immunization group. Passive immunization with TB mAbs may be of additional values as an intervention in specific circumstances, including malaria epidemic settings [48, 49].

Transmission-blocking strategies require improved knowledge of the basic biology of the parasite [50]. Recent efforts in genomics, transcriptomics and proteomics have revealed a large number of molecules that may play key roles in ookinete development [51–53]. Further, screening for novel vaccine candidates based on gene KO and phenotypic analysis will undoubtedly yield valuable information regarding the cell biology of the ookinetes [52]. Several ookinete proteins which play various roles in midgut colonization have been characterized, including the GPI-anchored P25 and P28 proteins [20, 54], circumsporozoite TRAP-related protein (CTRP) [55, 56], *Plasmodium* von Willebrand factor A domain-related protein (WARP) [57], secreted ookinete apical protein (SOAP) [55, 58], and the recently described putative secreted ookinete proteins (PSOPs) [52]. Here, we generated a Δ*psop25* line, and detected slightly reduced exflagellation activity of male gametocytes, but significantly reduced ookinete conversion rate *in vitro*. Whereas most Δ*psop25* parasites progressed normally to zygote and retort stages, further maturation to ookinetes was retarded, which resulted in a 60.9% reduction in the number of ookinetes as compared to the WT parasite. This phenotype shows some similarity with that of *psop2* knockout parasites, which appeared morphologically normal but showed reduced *in vivo* ookinete numbers [52]. The blockade in ookinete maturation in the Δ*psop25* parasite was further reflected in the reduction of oocyst density in DFA. The oocysts per mosquito midgut in those fed on the Δ*psop25* parasites was reduced by 69.4%, like that with the Δ*psop9* line [52]. Given that other PSOP proteins such as PSOP26 showed ookinete surface localization [26] and there is a possibility that these surface proteins interact, it would be interesting to determine whether *psop25* disruption could affect the expression of other PSOP proteins.

## Conclusions

This study confirmed that the full-length recombinant protein of a newly identified TBV candidate PSOP25 expressed in ookinetes of the rodent parasite *P. berghei* could also elicit a strong antibody response in mice. Both polyclonal mouse antisera and mAb against this protein recognized the surface of zygotes, retorts and ookinetes and possessed similar TB activities as the polyclonal antisera generated against the truncated version of this protein. Genetic KO study indicated that PSOP25 in *P. berghei* is required for ookinete formation and maturation. Collectively, PSOP25 is an excellent TBV candidate targeting the post-fertilization stages, and further assessment of TB activities in *P. falciparum* and *P. vivax* are warranted.

## Additional files

Additional file 1: Table S1. Primer information and sequences. (DOCX 15 kb)
Additional file 2: Figure S1. Predicted B cell epitopes of the PSOP25 protein (http://tools.iedb.org/bcell). Below is the protein domain architecture of PSOP25 with signal peptide highlighted in red, low complexity in pink, and transmembrane region in blue. **Figure S2.** The isotype of anti-rPSOP25 mAb was identified by ELISA using by the SBA Clonotyping™ System-HRP. The data represent two separate experiments. Error bar shows mean + standard deviation. **Figure S3.** Western blot analysis of *P. berghei* ookinete lysates with anti-rPSOP25 sera (PolyAb) and anti-rPSOP25 mAb. Lysates were subjected to electrophoresis under non-reducing conditions by SDS-PAGE. Pbs21 mAb was used as positive control; a control mouse serum (PBS) was used as negative control. (ZIP 2413 kb)

## Abbreviations

CTRP: Circumsporozoite TRAP-related protein
ELISA: Enzyme-linked immunosorbent assay
GPI: Glycosyl-phosphatidylinositol
i.p.: Intraperitoneally
IFA: Indirect immunofluorescence assay
iRBCs: Infected red blood cells
KO: Knockout
mAb: Monoclonal antibody
PBS: Phosphate-buffered saline
PBS-T: 0.05% Tween 20 in phosphate-buffered saline
PSOPs: Putative secreted ookinete proteins
RBCs: Red blood cells
SOAP: Secreted ookinete apical protein
TBS-T: 0.1% Tween 20 in Tris-buffered saline
TBV: Transmission-blocking vaccine
WARP: von Willebrand factor A domain-related protein
WT: Wildtype
