# Supplementary material for: Functional characterization of Plasmodium berghei PSOP25 during ookinete development and as a malaria transmission-blocking vaccine candidate
Source: Parasit Vectors. 2017 Jan 5;10:8. doi: 10.1186/s13071-016-1932-4 (PMC5217559; doi:10.1186/s13071-016-1932-4)
Supplement: Additional file 1: Table S1. — Primer information and sequences. (DOCX 15 kb) [file 13071_2016_1932_MOESM1_ESM.docx]

**Table S1** Primer information and sequences.

| Purpose | Primer name | Sequence |
| --- | --- | --- |
| Expression of rPSOP25 | *psop25*-F | CGCCATATGTCTGGTAATACAGGTGAAGTAGTACAAGGC  CA |
|  | *psop25*-R | CCCAAGCTTTTACAAAATATAAATGATAACTGC |
| Δ*psop25* line integration-specific PCR | QCR1 | AGCACAAGGGTCGTTTGAAGTCT |
|  | QCR1 | TGGTCCGAACAAACTTGACA |
|  | GT | TGTGCACGTGCGTTAGTGGGA |
|  | GW1 | - CATACTAGCCATTTTATGTG3 |
|  | GW2 | - CTTTGGTGACAGATACTAC |
|  | hDHFR-F | ATGGTTGGTTCGCTAAACTG |
|  | hDHFR-R | CTCCGCTAGCATCATTCTTCTC |
