# Supplementary figures and images for: Functional characterization of Plasmodium berghei PSOP25 during ookinete development and as a malaria transmission-blocking vaccine candidate
Source: Parasit Vectors. 2017 Jan 5;10:8. doi: 10.1186/s13071-016-1932-4 (PMC5217559; doi:10.1186/s13071-016-1932-4)

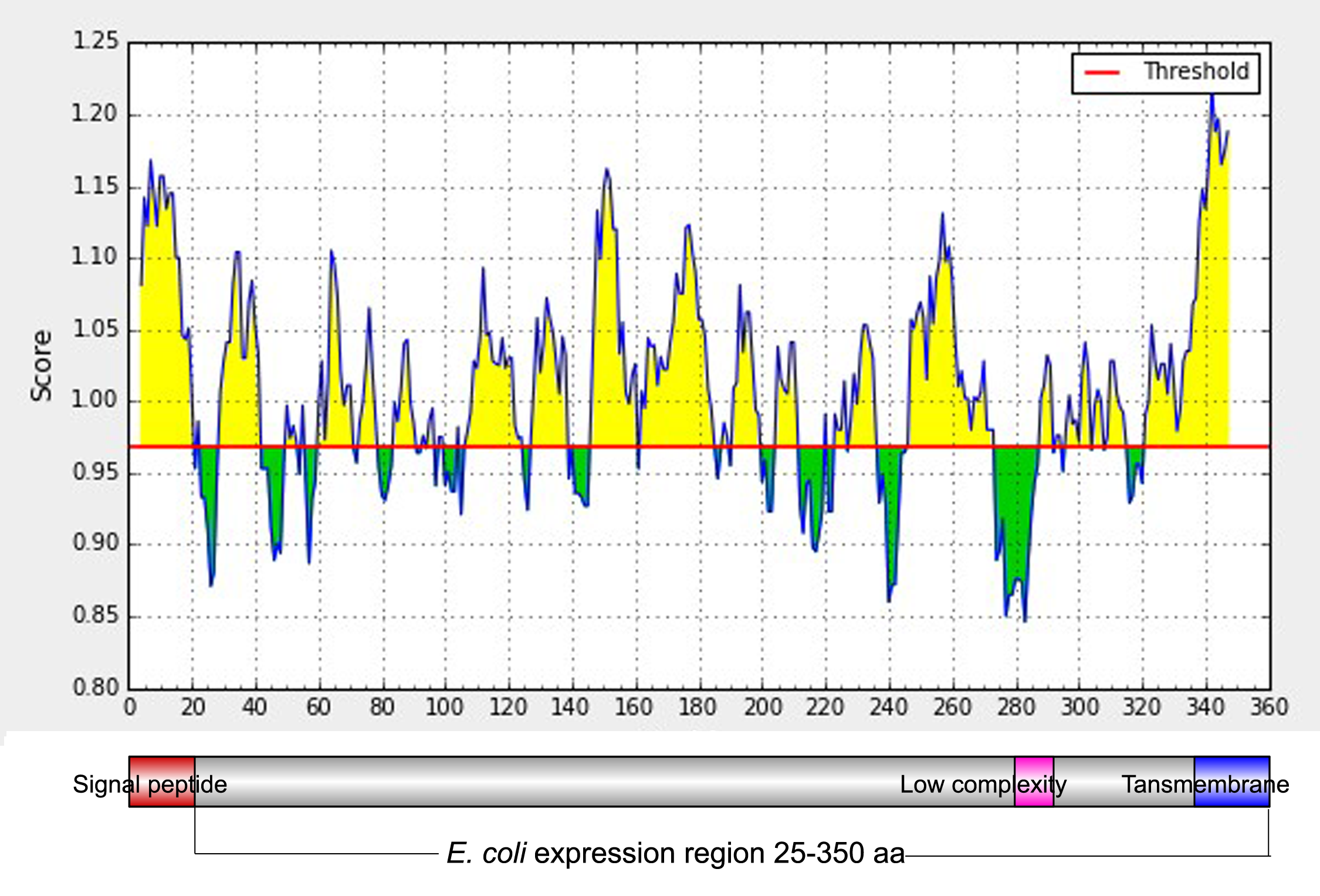

Supplement: Additional file 2: Figure S1. — Predicted B cell epitopes of the PSOP25 protein (http://tools.iedb.org/bcell). Below is the protein domain architecture of PSOP25 with signal peptide highlighted in red, low complexity in pink, and transmembrane region in blue. Figure S2. The isotype of anti-rPSOP25 mAb was identified by ELISA using by the SBA Clonotyping™ System-HRP. The data represent two separate experiments. Error bar shows mean + standard deviation. Figure S3. Western blot analysis of P. berghei ookinete lysates with anti-rPSOP25 sera (PolyAb) and anti-rPSOP25 mAb. Lysates were subjected to electrophoresis under non-reducing conditions by SDS-PAGE. Pbs21 mAb was used as positive control; a control mouse serum (PBS) was used as negative control. (ZIP 2413 kb) [file 13071_2016_1932_MOESM2_ESM.zip › Additional file 2-Figure S1.tif]

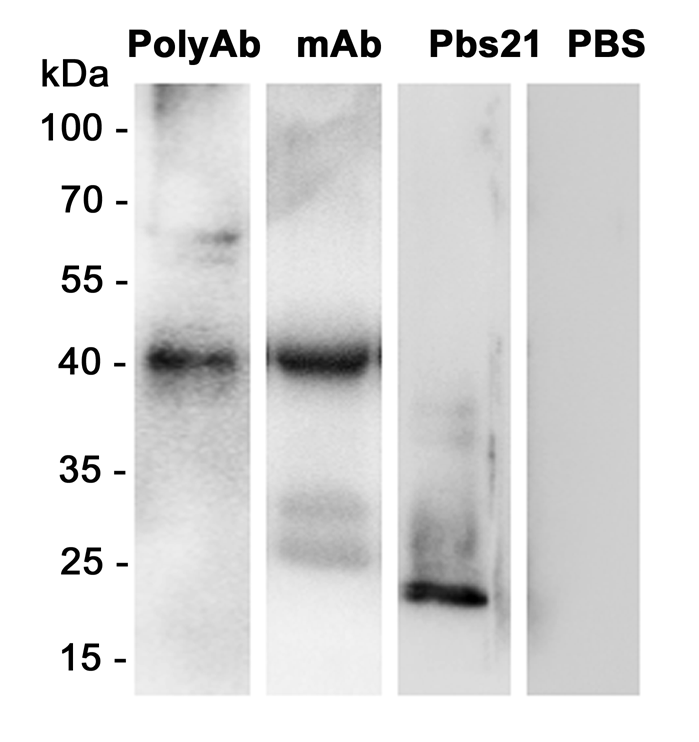

Supplement: Additional file 2: Figure S1. — Predicted B cell epitopes of the PSOP25 protein (http://tools.iedb.org/bcell). Below is the protein domain architecture of PSOP25 with signal peptide highlighted in red, low complexity in pink, and transmembrane region in blue. Figure S2. The isotype of anti-rPSOP25 mAb was identified by ELISA using by the SBA Clonotyping™ System-HRP. The data represent two separate experiments. Error bar shows mean + standard deviation. Figure S3. Western blot analysis of P. berghei ookinete lysates with anti-rPSOP25 sera (PolyAb) and anti-rPSOP25 mAb. Lysates were subjected to electrophoresis under non-reducing conditions by SDS-PAGE. Pbs21 mAb was used as positive control; a control mouse serum (PBS) was used as negative control. (ZIP 2413 kb) [file 13071_2016_1932_MOESM2_ESM.zip › Additional file 2-Figure S3.tif]
